# Epigenetic Regulation of Modulatory Neurotransmitter System Integrity in the Aging Brain: A Scoping Review Across the Lifespan

**DOI:** 10.3390/life16071122

**Published:** 2026-07-05

**Authors:** Khalid W. Freij, Arshiya Akbar, Philemon Domoyeri, Nunaya Polycarp, Dylan R. Higginbotham, Itika Arora, Edwin N. Aroke

**Affiliations:** 1 Department of Acute, Chronic & Continuing Care, School of Nursing, The University of Alabama at Birmingham, Birmingham, AL 35294, USA; kfreij95@uab.edu (K.W.F.); domoyeri@uab.edu (P.D.); 2 College of Medicine, Alfaisal University, Riyadh 11211, Saudi Arabiaiarora@alfaisal.edu (I.A.); 3 Department of Biology, College of Arts and Sciences, The University of Alabama at Birmingham, Birmingham, AL 35294, USA; npolycar@uab.edu (N.P.); drhiggin@uab.edu (D.R.H.); 4 Center for Biotechnology, Khalifa University of Science and Technology, Abu Dhabi P.O. Box 127788, United Arab Emirates; 5 Department of Public Health and Epidemiology, Khalifa University for Science and Technology, Abu Dhabi P.O. Box 127788, United Arab Emirates

**Keywords:** epigenetic aging, epigenetic clocks, DNA methylation, cerebral cortex, neuromodulatory neurotransmitter systems, brain aging, lifespan, DunedinPACE

## Abstract

Age-related changes in neurotransmitter systems contribute to declines in cognitive, emotional, and motor function, yet the biological mechanisms linking these changes to aging are not completely understood. Epigenetic regulation offers a promising framework to bridge this gap. DNA methylation-based biomarkers of biological aging (i.e., epigenetic clocks) capture cumulative and dynamic aspects of biological aging that may reflect vulnerability in neural systems beyond chronological age. However, whether these indices track with the integrity of neurotransmitter systems has not been systematically examined. This scoping review synthesizes evidence across human studies to evaluate how epigenetic aging processes influence neurotransmitter gene regulation and system function across the lifespan. We included 109 studies spanning 2005–2026. GABAergic genes (*GAD1*, *GABRA2*) showed the most consistent and reproducible age-related promoter hypermethylation across the cortex, inversely correlated with mRNA expression and corroborated by MRS evidence of cortical GABA decline. Dopaminergic and serotonergic evidence during normative aging was sparse; most epigenetic data in these systems came from disease cohorts. Histone modifications converged on neurotransmission and synaptic-plasticity loci, predominantly in Alzheimer’s disease tissue. Subcortical and brainstem nuclei central to monoaminergic and cholinergic systems remain under-investigated for normative aging epigenetic processes. Environmental and social determinants, socioeconomic status, childhood adversity, and chronic stress, were consistently associated with accelerated peripheral epigenetic aging, but brain-specific data are scarce.

## 1. Introduction

The cerebral cortex is sculpted during development by modulatory neurotransmitter systems (acetylcholine, dopamine, norepinephrine, and serotonin) that shape neurogenesis, neuronal migration, dendritic arborization, and the establishment of cortical circuits that subserve cognition, mood, and motor control throughout life [1]. The same modulatory systems must then be maintained across decades. Their cumulative dysregulation in mid- and late life is read out clinically as the cardinal features of dementia, late-life depression, and Parkinson’s disease, where cholinergic, monoaminergic, and noradrenergic deficits define the pathophysiology, and at the population level as a rapidly accelerating share of the global burden of disease, with more than 52.5 million adults aged ≥60 years currently affected by Alzheimer’s disease (AD) and other dementias and a near-fourfold increase projected by 2050 [2,3,4]. Yet the molecular processes that connect early cortical morphogenesis with later-life modulatory-system decline, and that determine which individuals age resiliently versus prematurely, remain incompletely mapped. Chronological age explains only a fraction of this variance [5]; biological age, indexed by the methylome and chromatin landscape that regulates neurotransmitter gene expression, is increasingly the more informative substrate [6].

Epigenetic mechanisms (DNA methylation, histone modifications, chromatin remodeling, and noncoding RNAs) are defined as molecular processes that regulate gene expression without altering DNA sequence. These mechanisms establish cell-type-specific gene-expression programs during cortical neurodevelopment and continue to shape neurotransmitter signaling throughout life [7]. They are distinct from upstream biological, environmental, and social factors, including stress, hormone signaling, nutrition, early-life exposures, and socioeconomic factors, which may modulate the epigenome across the lifespan. These factors are considered within the scope of the review only when evidence links them to age-related epigenetic alterations affecting modulatory neurotransmitter systems. Activity-dependent epigenetic regulation links neurotransmitter signaling to gene expression in ways that extend well beyond development. For example, histone serotonylation couples serotonergic activity to the transcription of GABAergic biosynthetic genes in the adult brain [8]. Across the lifespan, drift in regulatory marks at neurotransmitter biosynthetic enzymes, vesicular packaging machinery, and reuptake transporters offers a candidate explanation for the gradual functional erosion of cortical modulatory systems.

Recent advances in DNA methylation–based epigenetic clocks have provided quantitative measures of biological aging that complement traditional assessments of chronological age. First-generation clocks (Horvath, Hannum) predict chronological age [9,10]; second-generation clocks (PhenoAge, GrimAge) predict morbidity and mortality independently of chronological age [11,12]; and third-generation pace-of-aging measures (DunedinPACE) quantify the rate of biological aging rather than its accumulation [13]. Accelerated epigenetic aging has been associated with structural and functional brain changes, suggesting that biological age may provide insights into age-related alterations in neurotransmitter-system integrity [14]. Together, they offer a framework for examining whether cortical modulatory neurotransmitter systems age differently from the rest of the body and how age-related changes in regulatory architecture established during development contribute to this process.

Whether epigenetic aging signatures track with the integrity of specific modulatory neurotransmitter systems, and how the regulatory architecture established during cortical development is reshaped across the lifespan, has not been systematically mapped. The cholinergic system, central to cortical attention and declarative memory and a primary substrate of AD pathology, is regulated by methylation and chromatin modifications at *CHAT* and *ACHE* and at acetylcholine receptor loci, but normative aging epigenetic data in the cortex remain sparse. Dopaminergic transmission, essential for prefrontal executive function, working memory, and reward processing, is shaped by chromatin and methylation control at *SLC6A3* (DAT), *TH*, and *DRD2*, with disease-relevant evidence outnumbering normative aging coverage [15]. Noradrenergic signaling from the locus coeruleus modulates cortical arousal and cognitive control and is increasingly implicated in earliest-stage AD neuropathology, yet epigenetic studies of normative aging in this system are scarce. Serotonergic tone, regulated through methylation of *SLC6A4* and *TPH2*, contributes to late-life affective and cognitive trajectories [15,16].

Existing reviews categorize epigenetic clocks as global indicators of brain health and disease [17]. However, systematic reviews report inconsistent or modest associations between clocks and cognitive performance in dementia and mild cognitive impairment [18]. Disease-focused reviews have compiled clock performance data for AD, Parkinson’s, amyotrophic lateral sclerosis (ALS), and Huntington’s disease, but they have not integrated the modulatory neurotransmitter mechanisms that influence their respective phenotypes [19]. While some integrative perspectives suggest combining brain and biological age biomarkers, they fall short of providing a comprehensive system-level mapping [20]. To date, no review has combined findings on lifespan, cortical structure, and modulatory systems as current evidence now allows.

This scoping review aims to address this gap by systematically mapping how epigenetic aging processes alter neurotransmitter gene regulation in the human brain and the functional consequences for cognition, emotional regulation, and neurological diseases. Specifically, we will: (1) characterize system-specificity of age-related epigenetic changes across major neurotransmitter pathways, including evidence from epigenetic clock frameworks, histone modifications, and DNA methylation studies in cortical and subcortical regions; (2) evaluate the extent to which epigenetic biomarkers may predict neurotransmitter system decline prior to functional impairment, including their neuroimaging correlates and cross-tissue validity; and (3) examine biological, environmental, and social factors that influence epigenetic aging trajectories, examine methodological challenges, and delineate priority knowledge gaps to inform future clinical and translational research.

## 2. Methods

This review was reported in accordance with the Preferred Reporting Items for Systematic Reviews and Meta-Analyses extension for Scoping Reviews (PRISMA-ScR) guidelines [21]. The review was not prospectively registered. We searched the following databases: EMBASE [22], PubMed [23], and PsycInfo [24] for studies conducted from 1 January 2005 to 30 November 2025. Full search strategies including all Boolean operators, MeSH terms, controlled vocabulary, and date limits applied within each database are presented in Table 1. Search strings were adapted to the syntax and controlled vocabulary of each database, which creates differences across EMBASE, PubMed, and PsycINFO, as is standard practice. No additional filters beyond date range and English-language abstract availability were applied. The search strategy was intentionally broad to capture the intersection of aging, epigenetics, and neurobiology across the lifespan. To answer our review aims, additional eligibility criteria were applied during screening to identify studies specifically addressing epigenetic regulation of neurotransmitter systems. Formal risk of bias assessment of individual studies was not conducted, as this is consistent with the methodology of scoping reviews, which aim to map the breadth of available evidence rather than evaluate its quality [21].

### 2.1. Study Screening

**Figure 1:**
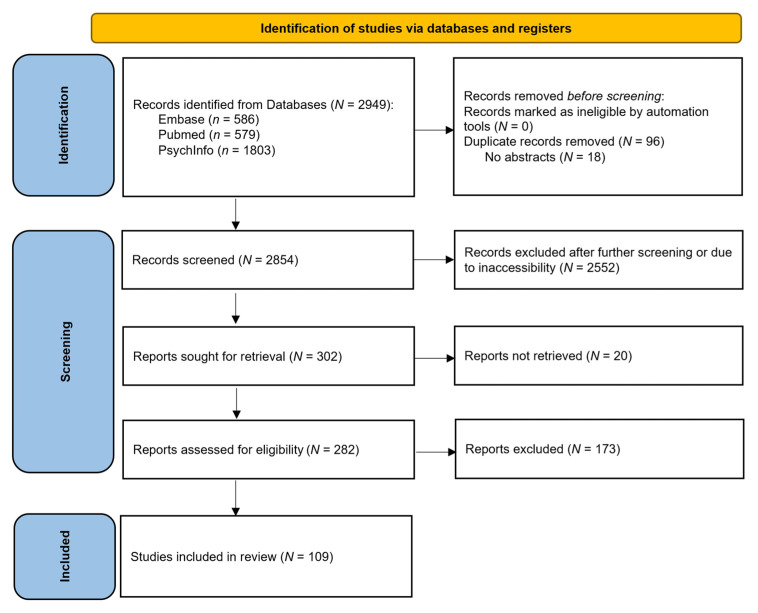
PRISMA flow diagram of study identification and selection.

After the initial database search, the available data were de-duplicated. KWF and PD independently reviewed the full-text manuscripts of the studies obtained for eligibility, and the lists were combined into a single list. ENA arbitrated between the authors’ split decisions. Studies were eligible for inclusion if they: (1) were conducted in human participants across any age range; (2) examined epigenetic mechanisms (including DNA methylation, histone modifications, or epigenetic clock measures) in relation to brain aging, cortical integrity, or neurotransmitter system function; (3) were published in peer-reviewed journals between 1 January 2005 and 30 November 2025; and (4) included an abstract available in English. Studies focused exclusively on non-human model organisms were excluded and will be addressed in a separate review. Review articles were excluded except where a review represented the sole available evidence for a specific domain addressed by the aims of the review. Additional exclusion criteria are summarized in Table 2. A PRISMA flow diagram depicting the selection process is presented in Figure 1. Database searches yielded 2949 records (Embase *n* = 586, PubMed *n* = 579, PsycInfo *n* = 1803). After removal of 96 duplicates and 18 records lacking abstracts, 2854 records were screened at title and abstract. Of these, 2552 were excluded and 302 were sought for full-text retrieval; 20 could not be retrieved, leaving 282 reports assessed for eligibility. A further 173 reports were excluded at full text, yielding a final corpus of 109 included studies.

To evaluate the sensitivity of the search strategy, a post hoc comparison was conducted in PubMed by removing the species/model organism block and the brain function/cognitive outcomes block from the search string. This yielded 960 records compared to 560 records with both blocks retained. The additional 400 records comprised studies related to non-brain epigenetic mechanisms in animal models, conference abstract compilations, and studies of peripheral organ systems with no relevance to neurotransmitter system aging in the human brain. However, a minority was found to be directly relevant to neuroepigenetic mechanisms of brain aging. These included studies of neuronal and cortical aging not captured by specific search terms, investigations of neurotransmitter system regulation across aging, and mechanistic epigenetic studies with indirect relevance to brain function. Importantly, the species/model organism block did not limit the search but instead retained relevant model organism research while reducing the overrepresentation of non-comparable biological systems to humans. The applied strategy preserved translational relevance without unnecessary loss of scope. This distribution underscores that the broader search increased sensitivity at the cost of specificity, whereas the refined strategy more effectively captured conceptually aligned literature.

### 2.2. Data Extraction and Analysis

The primary outcome of interest was the nature, extent, and distribution of evidence linking epigenetic clock measures to modulatory neurotransmitter system integrity and cortical aging across the human lifespan. Full-text articles were downloaded and processed using the AI-based text-mining tool, Microsoft 365 Copilot (Microsoft Corporation, Redmond, WA, USA), which was used specifically to support structured data extraction into a standardized table. For each included study, the following variables were extracted: authors, publication year, country, study design, diagnostic or classification criteria, genomic focus, significant genes or epigenetic marks, main findings, limitations, and future directions. Extraction was AI-assisted as Microsoft 365 Copilot outputs were reviewed by KWF and PD for each study, with discrepancies resolved through mediation by a third author. In addition, a random subset of 40% of entries underwent independent manual verification against the original full text to confirm accuracy and consistency. Microsoft 365 Copilot was not used for study selection, quality assessment, or synthesis. Because included studies varied widely in design, population characteristics, and outcome measures, we used a narrative synthesis approach rather than meta-analysis. Studies were grouped into thematic categories based on the literature: (1) Epigenetic Clock Frameworks and Brain Aging; (2) Histone Modifications in the Aging Brain; (3) Neurotransmitter System-Specific Epigenetic Changes; (4) Environmental and Social Determinants; (5) Neuroimaging Correlates; (6) Methodological Approaches and Challenges; and (7) Interpretive Challenges and Causal Inference. We then synthesized findings across categories to identify system-specific patterns, regional specificity, methylation-expression correlations, genetic modulation, and points of convergence across studies. No meta-analysis, meta-regression, or sensitivity analyses were conducted. All schematic and conceptual figures presented in this manuscript were created using BioRender (BioRender.com), a web-based platform designed for the creation of professional biomedical illustrations. Figures were exported under an academic publication license, consistent with BioRender’s guidelines for use in peer-reviewed publications.

## 3. Results

A post hoc sensitivity analysis comparing the refined PubMed search strategy with a broader version excluding species and brain-function-related term blocks yielded 560 and 960 records, respectively. Screening of the additional 400 records identified through the unrestricted search indicated that the majority were not directly relevant to the objectives of this review, consisting primarily of general molecular studies, non-central nervous system tissues, or systemic processes (~70%). A minority of records (~30%) demonstrated some degree of brain or neuronal relevance; however, only a subset met full inclusion criteria upon further screening. Collectively, this analysis demonstrates that the inclusion of the species/model organism block and the brain function/cognitive outcomes block improved the precision and interpretability of the evidence base without resulting in a meaningful loss of relevant literature.

The literature search identified references spanning 2005 to 2025, with the majority published after 2012. Included sources comprised a heterogeneous body of evidence encompassing epigenome-wide association studies, post-mortem brain analyses, neuroimaging studies using positron emission tomography (PET) and functional MRI (fMRI), cross-tissue concordance analyses, single-nucleus and cell-sorting studies, methodological validation papers, and longitudinal cohort studies examining epigenetic aging in peripheral tissue. Studies were conducted predominantly in European-descent populations, with limited representation of other racial and ethnic groups, and sampled a wide age range from fetal development through late life. Brain tissue analyses were concentrated in cortical regions, particularly the prefrontal and temporal cortex, with substantially less coverage of subcortical structures relevant to major neurotransmitter systems. The evidence was organized into six thematic domains: epigenetic clocks and biological aging; histone modifications in the aging brain; neurotransmitter system-specific epigenetic changes; neuroimaging of neurotransmitter systems; environmental and social determinants of epigenetic aging; and methodological approaches and cellular heterogeneity.

Across these domains, the epigenetic clock literature was anchored by ten methodological and validation studies establishing pan-tissue, blood-based, brain-specific, and pace-of-aging indices, collectively demonstrating that biological aging in neural tissue is regionally heterogeneous and measurable through DNA methylation signatures, though their direct application to neurotransmitter system integrity remains largely untested [9,10,11,12,13,14,25,26,27,28]. Histone modification evidence, drawn from six studies concentrated in AD rather than normative aging, consistently identified redistribution of activating and repressive marks at neurotransmission and synaptic plasticity loci [29,30,31,32,33,34]; however, the scarcity of normative aging data and the cortical sampling bias limit inference of the relationship between histone modifications and brain aging. The nineteen studies addressing neurotransmitter system-specific epigenetic changes were markedly asymmetric, with GABAergic genes, particularly *GAD1* and *GABRA2*, providing the most consistent evidence [35,36,37,38,39,40]. Dopaminergic evidence was largely confined to Parkinson’s disease rather than normative aging [41,42]; serotonergic findings were inconsistent and predominantly peripheral [43,44,45,46,47]; and glutamatergic evidence derived mainly from histone rather than DNA methylation studies [34,41,42,48,49]. Seven neuroimaging studies using PET and fMRI characterized in vivo neurotransmitter dynamics and their cognitive correlates across aging but remained largely unintegrated with molecular epigenetic data [50,51,52,53,54,55,56]. Thirteen studies examined environmental and social determinants of epigenetic clock acceleration, identifying consistent associations with socioeconomic disadvantage, childhood adversity, and chronic stress, though this cluster was notably disconnected from brain-specific neurotransmitter outcomes [57,58,59,60,61,62,63,64,65,66,67,68,69]. Eleven methodological studies identified brain cellular heterogeneity as a central interpretive challenge, with cell-type-specific approaches including fluorescence-activated nuclear sorting and single-nucleus methylation profiling revealing age-associated patterns invisible in bulk tissue analyses and cross-tissue concordance between blood and brain methylation only partial [70,71,72,73,74,75,76,77,78,79,80].

The results below organize the human evidence into eight thematic domains: epigenetic clocks and brain aging, chromatin context, histone modifications, system-specific neurotransmitter findings, environmental and social determinants, neuroimaging, methodological approaches, and interpretive challenges.

### 3.1. Overview

**Figure 2:**
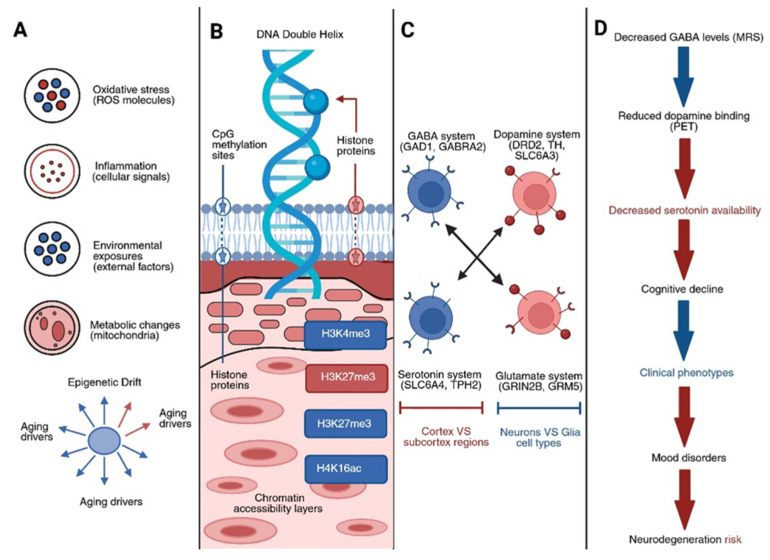
Aging-related epigenetic changes, neurotransmitter imbalance, and clinical outcomes. (**A**) Key aging-related drivers are illustrated, including oxidative stress with reactive oxygen species, inflammation with cellular signaling molecules, environmental exposures, and metabolic or mitochondrial changes. Together, these processes are summarized as contributors to epigenetic drift, represented by a central node influenced by multiple aging drivers. (**B**) Aging-related factors are depicted acting on the DNA double helix in neural cells. CpG methylation sites and surrounding histone proteins are shown as targets of these influences. Changes in CpG DNA methylation and histone marks (H3K4me3, H3K27me3, H4K16ac) remodel successive chromatin accessibility layers. This remodeling alters transcription of neurotransmitter genes. (**C**) Neurotransmitter systems are depicted as interacting neural units. The GABA system (*GAD1*, *GABRA2*) and dopamine system (*DRD2*, *TH*, *SLC6A3*) are shown with bidirectional arrows to indicate mutual influence. The serotonin (*SLC6A4*, *TPH2*) and glutamate (*GRIN2B*, *GRM5*) systems are also represented in this network. The panel indicates that these epigenetically regulated pathways can differ between cortical and subcortical regions and between neuronal and glial cell populations. (**D**) The panel shows a stepwise sequence of changes. First, GABA levels are reduced on magnetic resonance spectroscopy (MRS). Second, dopamine binding is lower on positron emission tomography (PET). Third, serotonin availability decreases. Together, these neurochemical shifts are depicted as giving rise to clinical phenotypes, mood disorders, increased susceptibility to neurodegeneration, and cognitive decline at the end of the trajectory.

Linking epigenetic aging processes to neurotransmitter system function requires a framework that connects epigenetic modifications through neurotransmitter gene expression, synaptic function and clinical outcomes. A conceptual framework organizing the evidence reviewed in subsequent sections is provided (Figure 2). Key epigenetic modifications include DNA methylation and histone modifications that alter chromatin accessibility. The four neurotransmitter systems are examined with respect to cortical and subcortical regional distinctions and between neuronal and glial types. These molecular and systems-level changes have measurable functional consequences that manifest clinically as cognitive decline, mood disorders, and increased neurodegeneration risk [51,55].

### 3.2. Epigenetic Clock Frameworks and Brain Aging

#### 3.2.1. DNA Methylation-Based Age Prediction

Epigenetic clocks use age-related changes in DNA methylation at specific CpG sites to estimate the biological age (Table 3). Horvath’s pan-tissue clock was developed using more than 8000 samples from 51 different tissue types and identified 353 CpG sites, many of which are located near Polycomb target genes and developmental regulators [10]. The blood-based Hannum clock, which includes 71 CpG sites, showed that genetic factors and sex can influence the rate of aging [9].

To address the consistent underestimation of age in older cortical samples, brain-specific models have been developed. For example, DNAmClockCortical significantly improved age prediction in the prefrontal, temporal, entorhinal, and cerebellar cortices of individuals older than 60 years [27].

Weighted gene co-expression network analysis of 2442 methylation arrays identified consensus age-associated modules enriched for CpG islands and neurogenesis genes, with remarkable preservation across independent datasets [81]. Regional heterogeneity is pronounced; the cerebellum exhibits slower epigenetic aging than cortical regions, with DNAm age typically 15 years younger than chronological age [82]. A large-scale epigenome-wide association study (EWAS) meta-analysis (1453 individuals) identified 236 significant CpGs in the prefrontal cortex versus zero in the cerebellum, despite >500 cerebellar samples, underscoring regional specificity [83]. In addition to chronological age predictors, newer algorithms, such as DunedinPACE, quantify the pace of biological aging rather than cumulative age, capturing dynamic aging processes associated with functional decline and mortality risk [13]. The neural correlates are concrete and cortex-relevant. Across the Dunedin, Framingham Heart Study Offspring, and Alzheimer’s Disease Neuroimaging Initiative cohorts (total N = 3380), faster DunedinPACE and higher GrimAge acceleration were the strongest, most consistent predictors of lower total brain volume, reduced hippocampal volume, thinner cortical thickness, and greater white-matter hyperintensity burden, outperforming first-generation clocks [28]. Diffusion magnetic resonance imaging (MRI) lesionometry has further linked GrimAge acceleration to spatially patterned cortical white-matter degeneration in healthy aging [26], and the imaging-derived counterpart Dunedin Pace of Aging Calculated from NeuroImaging (DunedinPACNI) predicts conversion to dementia, frailty, and mortality from a single MRI scan [14]. Others have suggested that the clocks are not interchangeable because each clock indexes distinct biological pathways [84]. Clock-specific deviations track socioeconomic and population-level factors that complicate direct comparison across cohorts [85]. Understanding where age-related methylation changes occur in the genome requires consideration of the underlying chromatin structure, as the regulatory significance of a methylation change depends partly on chromatin context.

#### 3.2.2. Chromatin Context of Age-Related Methylation

**Figure 3:**
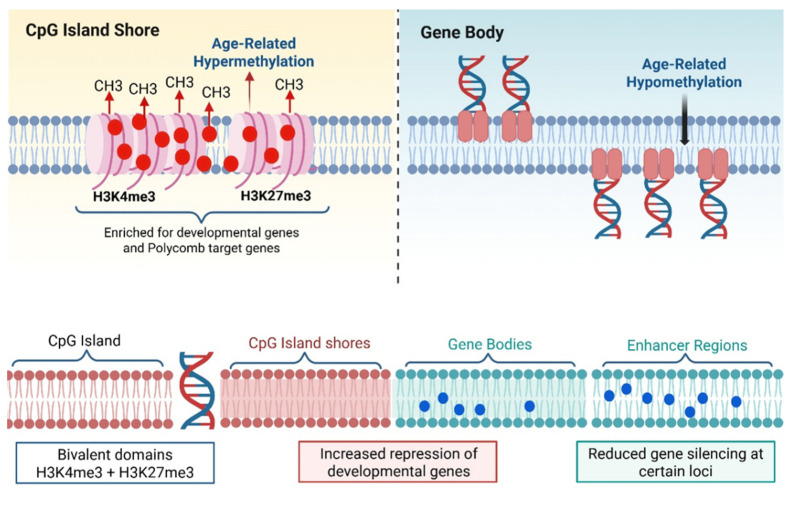
Region-specific patterns of age-related DNA methylation changes across the genomic landscape. (**Top left**): Age-related hypermethylation typically occurs at CpG island shores. These regions are enriched for developmental genes and Polycomb target genes and are often characterized by bivalent chromatin domains bearing both activating (H3K4me3) and repressive (H3K27me3) histone marks. (**Top right**): In contrast, age-related hypomethylation is more frequently observed within gene bodies. (**Bottom**): A summary schematic of the genomic landscape illustrating the functional consequences of aging-induced epigenetic remodeling across distinct regulatory regions: maintenance of bivalent domains at CpG islands, increased repression of developmental genes at CpG island shores, and reduced gene silencing at gene bodies and enhancer regions. Created with BioRender.com.

Age-related hypermethylation occurs primarily at CpG island shores and within bivalent chromatin domains marked by H3K4me3 and H3K27me3, whereas hypomethylation is more commonly observed in gene bodies and enhancer regions [86]. These distribution patterns suggest aging may be associated with both reduced gene silencing at certain loci and increased repression of genes involved in development-related processes. Cross-tissue analysis, including blood and three brain regions, in individuals aged 15–87 years identified many CpG sites that exhibited consistent age-related changes across tissues. However, it also revealed brain-specific methylation patterns, particularly in genes related to synaptic function [87]. Figure 3 illustrates the contrasting patterns of age-related DNA methylation changes at the CpG island shores and gene bodies. Beyond DNA methylation, chromatin structure is further regulated by histone post-translational modifications, which undergo their own age-related changes and interact with methylation patterns at shared genomic loci.

#### 3.2.3. Histone Modifications in Aging Brain

Histone post-translational modifications undergo age-related changes, although the literature on these modifications is less extensive than that on DNA methylation modifications. Validation studies have established that histone methylation marks (H3K4me3, H3K9me2/me3, H3K27me2/me3, and H3K36me3) remain stable in the postmortem brain for 48–72 h, while acetylation marks decline within 24 h [29,30].

##### Histone Acetylation

Genome-wide chromatin immunoprecipitation sequencing (ChIP-seq) profiling of H4K16 acetylation in the lateral temporal lobe revealed that normal aging appears to be characterized by H4K16ac enrichment at specific loci, whereas AD entails dramatic losses near aging- and pathogenesis-associated genes [31]. Genomic regions showing H4K16ac changes were associated with GWAS variants and expression quantitative trait loci (eQTLs). Integrated multi-omics (H3K27ac ChIP-seq, H3K9ac, transcriptomics, proteomics) showed that genes with altered acetylation were enriched for chromatin modification and transcriptional regulation functions [32].

##### Histone Methylation

ChIP-seq mapping of H3K4me3 (activating) and H3K27me3 (repressive) marks in the prefrontal cortex of patients with mild cognitive impairment and AD versus controls found pronounced H3K4me3 losses at promoters enriched for synaptic plasticity and neurotransmission, with gains at promoters of transcriptional regulators [33]. This redistribution pattern of the loss of activating marks at neuronal function genes and gain at regulatory genes appears to parallel DNA methylation observations.

Gene ontology analyses of loci showing age- or disease-related histone modification changes consistently suggest enrichment for neurotransmission, synaptic function, and neuronal signaling [31,33]. This suggests collective epigenetic remodelling of neurotransmitter-related gene sets. A structured overview of the major histone modifications implicated in neurotransmitter-related loci is summarized in Table 4. The histone modification changes are not uniformly distributed across the genome but are disproportionately concentrated at genes governing neurotransmission and synaptic function, motivating a closer examination of epigenetic changes within specific neurotransmitter systems.

### 3.3. Neurotransmitter System-Specific Epigenetic Changes

#### 3.3.1. GABAergic System

GABAergic genes show the most consistent age-related DNA methylation changes identified to date in the human cortex. Progressive *GAD1* promoter hypermethylation across the lifespan (fetal to 87 years) was inversely correlated with mRNA expression (R^2^ = 0.29–0.42) in the temporal cortex [38,39]. Numata et al. classified *GAD1* as “type 1”, a linear, progressive hypermethylation beginning in late childhood and continuing into old age [38]. *GABRA2* shows similar patterns, with mRNA levels inversely related to promoter methylation [38]. The key age-related DNA methylation findings across major neurotransmitter genes are summarized in Table 5.

Chromatin immunoprecipitation experiments separating H3K4me3-marked open chromatin from repressive chromatin showed that *GAD1* promoter methylation was enriched in the repressive fractions [37]. RNA sequencing further identified 10 novel GAD1 transcript isoforms, the expression of which was inversely correlated with that of full-length *GAD67*. In addition, methylation at specific CpG sites was associated with schizophrenia risk variants and *GAD1* expression in the dorsolateral prefrontal cortex [88].

**Figure 4:**
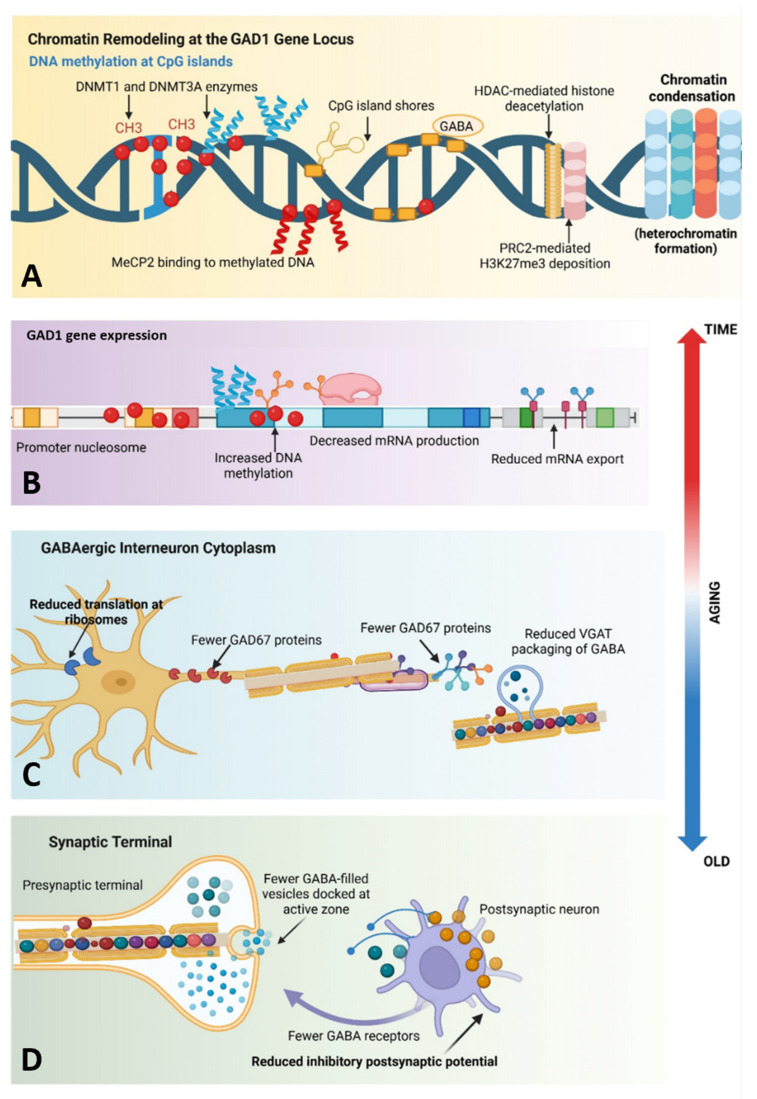
Overview of epigenetic mechanisms underlying age-related GABAergic dysfunction. (**A**) Aging promotes epigenetic remodeling at the *GAD1* gene locus through increased CpG methylation, MeCP2 binding, histone deacetylation, and PRC2-mediated H3K27me3 deposition, resulting in chromatin condensation and transcriptional repression. (**B**) Epigenetic silencing and transcriptional repression. Increased DNA methylation and chromatin remodeling are associated with reduced gene expression, characterized by decreased mRNA production and impaired mRNA export. (**C**) Reduced gene expression in GABAergic interneurons is associated with lower *GAD67* protein levels and impaired VGAT-mediated packaging of GABA into synaptic vesicles. (**D**) At the synapse, reduced GABA availability and fewer postsynaptic GABA receptors weaken inhibitory neurotransmission, leading to diminished inhibitory postsynaptic signaling. The vertical gradient indicates progression of these changes during aging. Created with BioRender.com.

These molecular findings are consistent with the functional observations. Magnetic resonance spectroscopy studies have reported lower GABA concentrations in the prefrontal and motor cortices of older adults than in those of younger individuals [35,40]. Postmortem analyses have reported reduced *GAD67* protein levels in the aged brain [36]. Interestingly, cognitively intact adults aged 85 years show relative stabilization of GABA levels, suggesting that preserved GABAergic function may differentiate successful aging from pathological aging [40]. Figure 4 illustrates the multi-level cascade from chromatin remodeling at the *GAD1* gene locus to reduce GABAergic synaptic transmission during aging. In contrast to the relatively robust GABAergic evidence, the epigenetic regulation of dopaminergic genes during normative aging is substantially less characterized.

#### 3.3.2. Dopaminergic System

Although dopamine neurotransmission shows evidence of functional decline with aging, direct evidence for age-related DNA methylation changes in dopaminergic genes during normative human brain aging remains limited. PET studies have consistently reported progressive reductions in striatal dopamine transporter availability, D2 receptor binding, and dopamine synthesis capacity in older adults [89,90]. However, corresponding epigenetic data on healthy aging are scarce.

Most epigenetic studies on dopaminergic genes have focused on Parkinson’s disease rather than normative aging. For example, hypomethylation of SNCA has been associated with increased gene expression in patients with Parkinson’s disease [42], and whole-genome bisulfite sequencing of induced pluripotent stem cell-derived dopaminergic neurons has shown global hypermethylation in this condition [41]. These disease-related alterations cannot be assumed to reflect the normal aging process. A comprehensive lifespan analysis of the prefrontal cortex identified *DRD2* among genes showing age-related methylation changes, but without detailed characterization [38]. Other key dopaminergic genes, including *TH*, *SLC6A3*, and *COMT*, have not been systematically evaluated. *SATB1* and *GBA* are genetic risk factors in a gene regulatory pathway that includes microRNA-22 (miR-22), and the dysregulation can lead to a cellular senescence-like phenotype in dopaminergic neurons through the upregulation of glucocerebrosides [91].

PET and fMRI have proven useful for evaluating and managing psychiatric disorders by assessing cerebral metabolism, blood flow, and neurotransmitter changes [52]. They can measure neurotransmitter levels in healthy and psychiatric conditions and evaluate the effects of treatment. Some studies have combined PET measurements of dopamine with fMRI measures of brain activation to link neurochemistry to neural activity during aging [50]. For example, PET studies have been used to evaluate altered dopamine modulation in schizophrenia and AD, suggesting that serotonin modulation of dopamine may be blunted in schizophrenia and that the dopamine system in patients with AD fails to compensate for changes in cholinergic function [53]. These methods enable in vivo mapping of connectivity patterns within and between neurotransmitter systems, such as serotonin and opioid systems [56]. Interpreting the molecular and neuroimaging evidence requires careful consideration of the methodological constraints that shape what can and cannot be inferred from existing studies.

This limited evidence likely reflects the methodological constraints. First, many brain methylation studies have focused on cortical regions, where dopaminergic cell bodies are relatively sparse, rather than midbrain regions such as the substantia nigra and ventral tegmental area. Second, bulk tissue methylation arrays may not be sensitive enough to detect changes specific to the small population of dopaminergic neurons. Third, without cell-type-specific approaches, methylation signals from dopaminergic neurons may be diluted by the presence of other cell types in the brain tissue [54,70]. Similar methodological constraints apply to the serotonergic system, where the available evidence is largely derived from peripheral tissue rather than brain-specific analyses.

#### 3.3.3. Serotonergic System

*SLC6A4* promoter methylation has been widely investigated in peripheral blood in relation to depression and stress [43,47], with studies examining brain tissue during normative aging remaining limited. In a cohort of community-dwelling adults aged 65 years and older, *SLC6A4* methylation showed a trend-level association with age (β = 0.23, *p* = 0.08). In the same study, the 5-HTTLPR genotype influenced the relationship between methylation and depression [47].

The relationship between peripheral *SLC6A4* methylation and serotonergic brain function remains unclear. Wang et al. reported that higher methylation at specific CpG sites in T cells and monocytes was associated with lower serotonin synthesis in the orbitofrontal cortex, as measured by PET [46]. In contrast, a larger study including 344 participants found no association between peripheral *SLC6A4* or *TPH2* methylation and brain serotonin transporter or receptor availability, as assessed by positron emission tomography [44]. These inconsistent findings suggest that the relationship may be context-dependent and raise questions about the reliability of peripheral tissue methylation as a proxy for brain serotonergic function.

A meta-analysis of *SLC6A4* methylation across psychiatric disorders reported substantial heterogeneity influenced by genetic variation, environmental exposures, developmental stage, and tissue type [43]. In addition, a lifespan study of the prefrontal cortex did not identify serotonergic genes among the loci most strongly associated with age, which may reflect limitations in array coverage or tissue sampling [38]. Whereas dopaminergic and serotonergic evidence is limited primarily by tissue accessibility and cross-tissue validity concerns, the gap in glutamatergic epigenetics reflects a different methodological asymmetry, where histone modifications are the primary subject rather than DNA methylation.

#### 3.3.4. Glutamatergic System

Current evidence on the epigenetic regulation of glutamatergic genes during aging is derived mainly from studies of histone modifications rather than DNA methylation. For example, histone H3K4me3 enrichment at the promoters of ionotropic glutamate receptors, including NMDA, AMPA, and kainite receptors, as well as metabotropic glutamate receptors, has shown developmental and region-specific patterns of expression. *GRIN2B* and *GRM5* display higher H3K4me3 levels in the cortex than in the cerebellum [34].

Epigenome-wide association studies of AD have identified *GRIA4*, which encodes the GluA4 subunit of the AMPA receptor, as a gene with altered methylation [48,49]. However, these analyses typically adjust for age to isolate the disease-related effects. Therefore, the specific contribution of age-related methylation changes in glutamate receptor genes to normative brain aging remains unclear and warrants further investigation. The system-specific epigenetic changes are not isolated, as environmental and social exposures across the lifespan modulate epigenetic mechanisms and the rate and trajectory of epigenetic aging, with potential downstream consequences for neurotransmitter system integrity.

#### 3.3.5. Neuroimaging Evidence Linking Epigenetics and Neurotransmission

PET and fMRI are valuable tools for studying neurotransmitter systems and their changes with aging in vivo [51,55]. Using PET, the neurotransmitter and neuroreceptor systems of the human brain can be localized in precise anatomical contexts, and several quantitative parameters of these systems can be measured [55]. These neuroimaging techniques are used to identify the neural correlates of age-related declines in episodic and working memory [55].

Dysfunctions in the dopamine, noradrenaline, and cholinergic systems are implicated in age-related cognitive decline, as evidenced by functional neuroimaging studies using PET and pharmacological fMRI [51,55]. PET and fMRI studies have contributed to understanding how brain activity during cognitive performance changes with age [51]. A key finding is that brain activity tends to be less lateralized in older adults than in younger adults, a pattern conceptualized in the Hemispheric Asymmetry Reduction in Older aDults (HAROLD) model [51].

### 3.4. Environmental and Social Determinants

In humans, the integration of social determinants with epigenetic aging and neurotransmitter function remains limited but is growing. Studies examining epigenetic clock acceleration have identified associations with socioeconomic status [57,63,68,69], childhood adversity [65,66], and chronic stress exposure [58,59,60,61,62]. Lower SES and experiences of discrimination have been associated with accelerated epigenetic aging in blood-derived DNA, as brain-specific analyses are not available. Early life adversity has been linked to altered DNA methylation in genes involved in stress response and neurotransmitter signaling, including serotonergic (e.g., *SLC6A4*, *HTR1A*) and dopaminergic (e.g., *DRD2*, *DAT1*) genes [64]. While most human studies have examined peripheral tissue or saliva, post-mortem brain studies suggest that childhood trauma is associated with persistent epigenetic modifications in the hippocampal regions [67], though direct links to age-related declines in neurotransmitters await investigation. While molecular epigenetic approaches characterize gene-level regulation, neuroimaging methods provide complementary in vivo evidence of neurotransmitter system function and its age-related changes.

### 3.5. Methodological Approaches and Challenges

#### 3.5.1. EWAS Design Considerations

Epigenome-wide association studies use array-based platforms such as the Illumina 450 K array, which measures approximately 450,000 CpG sites and covers approximately 1.5% of the genome, and the EPIC array, which includes approximately 850,000 CpG sites with roughly 3% genome coverage. These platforms are enriched for regulatory regions [78]. Because hundreds of thousands of CpG sites are tested simultaneously, a stringent correction for multiple comparisons is required. Common approaches include the Bonferroni correction, with significance thresholds around *p* < 1 × 10^−7^, and false discovery rate methods. Consequently, large sample sizes are necessary to achieve sufficient statistical power. Meta-analyses that combine effect sizes across cohorts using inverse-variance weighting can substantially improve power [83]. In addition, region-based analyses that aggregate signals across differentially methylated regions can increase detection sensitivity compared to single-CpG analyses [77].

A continuing challenge in EWAS is distinguishing methylation changes associated with normal aging from those associated with disease or terminal decline. Many studies adjust for chronological age to identify disease-specific effects, which removes the shared variance between age and disease. However, some age-related methylation changes may contribute directly to disease risk, and adjusting for age may exclude biologically relevant signals. Study designs that include multiple age groups and disease states can help clarify these relationships [11,25].

#### 3.5.2. Cellular Heterogeneity and Cell-Type-Specific Approaches

Brain cellular heterogeneity presents a major challenge for interpreting epigenetic findings. The human cortex contains excitatory neurons (~70–80% of neurons), GABAergic interneurons (~10–20% of neurons), and non-neuronal cell types, including astrocytes, oligodendrocytes, microglia, and vascular cells. Each of these cell types has a distinct epigenomic profile [75]. In addition, cell-type proportions change with age. Astrocyte and microglial populations tend to increase, while certain neuronal populations may decline [80,92]. If cellular composition is not considered, observed methylation differences may reflect shifts in cell-type proportions rather than intrinsic changes within specific cells.

Several computational methods have been developed to estimate cell-type proportions from bulk methylation data using such reference profiles. The Houseman method was initially developed for blood [74], and brain-specific adaptations have since been introduced [71]. Many epigenome-wide association studies now include estimated cell-type proportions as covariates, although these estimates are not fully accurate.

Physical cell sorting provides a more direct separation of cell types. Fluorescence-activated nuclear sorting using the NeuN antibody to distinguish neuronal from non-neuronal nuclei has identified numerous age-associated differentially methylated positions that are not detectable in bulk tissue analyses [70]. For example, *CLU*, *SYNJ2*, and *NCO*R2 showed neuron-specific age-related patterns, whereas *RAI1*, *CXXC5*, and *INPP5A* exhibited glia-specific ones. Notably, many methylation changes associated with AD have been observed predominantly in non-neuronal cells [70].

Single-nucleus approaches allow for even greater resolution. Single-nucleus RNA sequencing has identified multiple neuronal and glial subtypes in the human brain [73]. More recently, single-nucleus methylation methods, such as snmC-seq, have enabled the profiling of DNA methylation at the level of individual nuclei, enabling cell-type resolution based on neurotransmitter identity [76]. The application of these approaches could clarify whether changes, such as GAD1 methylation, are restricted to GABAergic interneurons or occur more broadly across cell populations.

#### 3.5.3. Cross-Tissue Concordance: Brain Versus Periphery

Multiple studies have directly compared methylation between the blood and brain. Correlation coefficients of 0.3–0.4 for age-associated CpGs highlight partial but incomplete concordance [87,93]. Approximately 7000 CpGs show correlated levels across tissues, whereas many are tissue-specific [93]. For neurotransmitter genes, concordance appears to be gene- and locus-specific. Peripheral *SLC6A4* methylation showed variable correlations with brain serotonin function across specific CpG sites and functional endpoints [44,46].

Reference epigenome projects highlight the strong tissue specificity of regulatory landscapes [94,95]. While peripheral tissues may provide useful biomarkers, they cannot fully substitute for brain tissue when investigating brain-specific aging processes. Beyond the technical challenges of measurement, the existing evidence base also raises broader questions of causal inference that bear directly on how findings from epigenetic aging studies should be interpreted.

### 3.6. Interpretive Challenges and Causal Inference

#### 3.6.1. Cross-Sectional Versus Longitudinal Designs

Cross-sectional studies identify associations at a single time point but cannot determine the direction of the effect. For example, it remains unclear whether increased *GAD1* promoter methylation leads to reduced gene expression and lower GABA levels or whether reduced transcriptional activity results in increased methylation through feedback mechanisms. Experimental manipulation of model systems can help address causality [96]; such approaches are not currently feasible in the human brain.

Mendelian randomization offers another strategy for examining causal relationships. This method uses genetic variants associated with DNA methylation, known as methylation quantitative trait loci (mQTLs), as instrumental variables [97]. If a genetic variant is associated with both methylation and an outcome, and the effect on the outcome occurs only through methylation, it supports a causal role. Mendelian randomization has been applied to test whether epigenetic age acceleration influences health outcomes, with mixed results [98]. However, analyses of mQTLs in brain tissues remain limited [99,100].

Longitudinal studies of peripheral tissues provide additional, albeit indirect, evidence. In blood samples collected over time, accelerated epigenetic aging was associated with an increased mortality risk after adjusting for baseline age [101]. Simultaneously, measures of epigenetic aging show only moderate stability within individuals over approximately seven years, with correlation coefficients ranging from 0.3 to 0.6, suggesting considerable intra-individual variation [101].

#### 3.6.2. Effect Sizes and Functional Significance

Many associations identified in epigenome-wide association studies involve small absolute differences in methylation, often in the range of 2–5% in beta values between different age groups [81]. The functional significance of these differences depends on their regulatory context. At some regulatory elements, even small changes in methylation may substantially affect transcription factor binding and gene expression, where threshold effects are present [102]. In other regions, similar changes may have little or no functional consequences. Integrating DNA methylation data with gene expression analyses, such as methylation-expression quantitative trait locus studies, may help identify loci with functional relevance [103].

The biological significance of epigenetic clocks remains under discussion. Proposed explanations include variations in the rate of epigenetic maintenance processes [10], the gradual accumulation of stochastic methylation changes over time, referred to as epigenetic drift [104], and the continued operation of developmental programs later in life [105]. These models differ in whether epigenetic clocks reflect the active drivers of aging, secondary consequences of other processes, or relatively neutral changes. Current evidence suggests that aspects of each of these mechanisms may contribute to this phenomenon.

## 4. Integration and Synthesis

Several overarching patterns emerge:

**System-specific patterns.** GABAergic genes show the most consistent age-related increases identified to date in DNA methylation in the cortex [38,39], in contrast to the sparse evidence for dopaminergic and serotonergic genes during normative aging. This may reflect true biological differences or technical factors (brain region sampling, cell-type heterogeneity, and array coverage).

**Regional specificity.** Cortical regions appear to show more rapid epigenetic aging than the cerebellum [82]. Within the cortex, the prefrontal and temporal regions are best characterized. Subcortical structures (substantia nigra, ventral tegmental area, raphe nuclei, and basal forebrain) remain understudied despite their importance in neurotransmitter systems.

**Partial methylation-expression correlation.** *GAD1* and *GABRA2* show inverse correlations (R^2^ = 0.29–0.42) [38,39], suggesting that methylation may explain only a fraction of the expression variance. Gene expression is influenced by multiple factors, including transcription factors, chromatin state, histone modifications, and three-dimensional (3D) organization beyond DNA methylation [106].

**Genetic modulation.** mQTL analyses have identified variants that influence methylation [99,100]. *SLC6A4* methylation is influenced by the 5-HTTLPR polymorphism [47], and *GAD1* methylation is associated with genetic risk variants [88]. Variants are associated with blood epigenetic aging rates, with TERT showing genome-wide significance [98].

**Figure 5:**
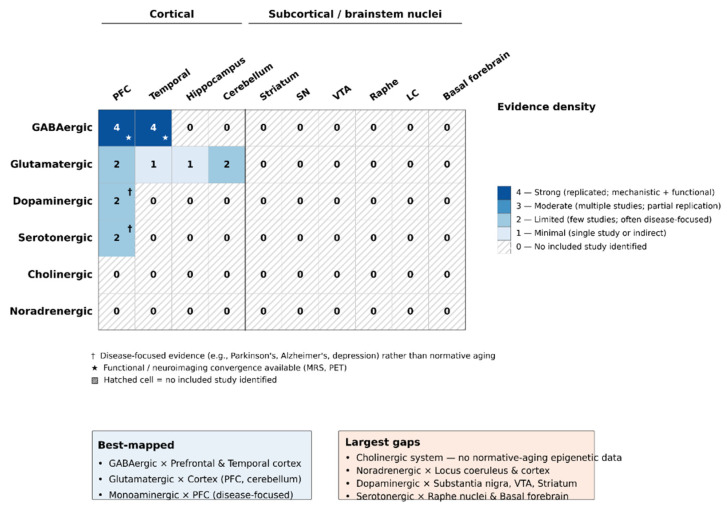
Evidence density for epigenetic regulation of neurotransmitter systems across cortical and subcortical regions of the human brain. Cells display the density of included evidence (*n* = 109 studies) for each neurotransmitter system × brain region combination, scored on a five-level ordinal scale from 0 (no included study identified) to 4 (multiple replicated studies with mechanistic and functional convergence). Rows are ordered by total evidence density (descending) and grouped into cortical (prefrontal cortex, temporal cortex, hippocampus, cerebellum) and subcortical/brainstem (striatum, substantia nigra, ventral tegmental area, raphe nuclei, locus coeruleus, basal forebrain) superclusters separated by a vertical rule. The figure illustrates two central observations of this synthesis: (i) the GABAergic and, to a lesser extent, glutamatergic systems in cortical regions are comparatively well-mapped, with replicated, mechanistically grounded evidence from independent cohorts; and (ii) the subcortical and brainstem nuclei central to monoaminergic and cholinergic neurotransmission remain substantially under-investigated for normative aging epigenetic processes. Companion summary boxes at the foot of the figure highlight the three best-mapped and four largest-gap cells.

**Convergence despite heterogeneity.** Despite methodological variations (brain regions, array platforms, age ranges, analytical approaches), there is modest convergence on certain core findings. *GAD1* hypermethylation has shown replication across independent cohorts [38,39]. Large EWAS meta-analyses successfully identified reproducible loci [83]. The evidence-density landscape across neurotransmitter systems and brain regions is summarized in Figure 5.

## 5. Future Directions and Translational Potential

### 5.1. Technical Advances

**Subcortical profiling.** Comprehensive epigenomic profiling of the substantia nigra, ventral tegmental area, striatum (dopamine), raphe nuclei (serotonin), and basal forebrain (acetylcholine) may help address cortical bias and clarify region-specific vulnerabilities.

**Multi-omic integration.** The integration of DNA methylation, 5-hydroxymethylcytosine, histone modifications, chromatin accessibility, and 3D architecture in the same samples may provide systems-level views [32].

**Single-nucleus resolution.** Simultaneous profiling of methylation, transcriptome, and chromatin accessibility at the single-nucleus level could enable neurotransmitter-defined cell-type resolution [76,107]. This could help determine whether *GAD1* methylation occurs within surviving GABAergic interneurons or reflects selective population loss.

### 5.2. Longitudinal and Intervention Studies

**Longitudinal designs.** Combining peripheral tissue epigenetic profiling with repeated neuroimaging (PET, MRS) and cognitive assessment could establish temporal relationships with limited brain-specific data in humans. If peripheral methylation signatures reliably predict brain neurotransmitter function [46], this could enable non-invasive monitoring of brain neurotransmitter function. Additionally, cumulative socioenvironmental exposures across the lifespan shape an individual’s epigenome. Longitudinal follow-up studies are therefore important to disentangle the relationships among socioenvironmental factors (both protective and adverse), brain function, and epigenomic trajectories over time.

**Genetic architecture.** Systematic brain tissue mQTL analyses would distinguish genetically influenced from environmentally responsive patterns. Understanding which neurotransmitter gene methylation changes are under genetic control versus those responsive to modifiable factors has implications for intervention [98,99].

**Intervention studies.** Testing whether lifestyle, pharmacological, or epigenetic editing approaches can slow epigenetic aging and improve neurotransmitter function has significant translational value. Small pilot studies suggest that lifestyle interventions can slow epigenetic aging [108,109], but whether this extends to brain-specific outcomes remains unknown and requires rigorous testing. The repurposing of drugs that modulate epigenetic machinery (DNMT inhibitors, HDAC inhibitors) could be evaluated using outcomes related to neurotransmitter function. Including more populations in these studies can also increase the generalizability of the results to better serve all individuals rather than a few populations.

### 5.3. Translational Implications

The central objective of this scoping review was to map the existing evidence on whether epigenetic dysregulation of neurotransmitter systems is associated with age-related cognitive decline, mood disorders, and neurodegenerative conditions, and to identify whether these mechanisms represent plausible targets for future therapeutic investigation. The available evidence suggests that epigenetic regulation remains dynamic throughout life, suggesting potential windows for intervention.

Among neurotransmitter systems, GABAergic genes demonstrate the most consistent age-related and epigenetic changes. These findings are consistent with reported reductions in GABA levels and age-related inhibitory dysfunction [35,40]. It remains to be established whether strategies aimed at preserving GABAergic gene expression through epigenetic modulation can help maintain cognitive function in later life.

In contrast, evidence for epigenetic changes in the dopaminergic and serotonergic systems during normative aging is limited, which prevents firm conclusions but does not exclude the possibility of meaningful regulation. Addressing this gap will require methodological improvements, particularly the use of cell-type-specific approaches in anatomically relevant brain regions.

The intersection of social determinants, epigenetic aging, and neurotransmitter dysfunction has profound implications for health equity in the field of cognitive aging. Populations experiencing chronic socioeconomic disadvantage, discrimination, or cumulative life stress may exhibit accelerated epigenetic aging in brain regions critical for neurotransmitter function, potentially contributing to disparities in cognitive decline, depression, and neurodegenerative disease risk. If environmental and social factors shape the epigenetic landscape of neurotransmitter systems, then interventions targeting these upstream determinants, such as stress reduction programs, nutritional support, social connection initiatives, or policies addressing structural inequities, may represent modifiable targets for promoting healthy brain aging at the population level.

### 5.4. Limitations

Several limitations should be acknowledged. First, the search strategy was originally developed for a broader review and was not re-run de novo for the present scoping question; relevant studies indexed after the original search date or outside its terms may have been missed. Second, data extraction was conducted using a generative AI tool (Microsoft 365 Copilot) with manual validation on a random subset of entries; although this approach increased efficiency, it may introduce reproducibility constraints because the underlying model is proprietary and may change over time. Third, the included literature is heavily weighted toward cortical regions, leaving subcortical structures central to monoaminergic and cholinergic systems (substantia nigra, ventral tegmental area, raphe nuclei, locus coeruleus, basal forebrain) under-represented. Fourth, most evidence on environmental and social determinants derives from peripheral tissue and may not generalize to brain epigenomes. Finally, the protocol was not prospectively registered. These limitations should be considered when interpreting the synthesis and inform the recommendations in Section 5.

## 6. Conclusions

In returning to the framework that Berger-Sweeney and Hohmann set out for modulatory neurotransmitter regulation of cortical morphogenesis, this synthesis suggests that the same systems they identified as developmentally pivotal, cholinergic, dopaminergic, noradrenergic, and serotonergic, are also the systems whose lifespan epigenetic regulation is least well charted in the aging cortex. Closing that gap is the principal research priority that emerges from this review.

Epigenetic mechanisms, including DNA methylation and histone modifications, appear to play a role in regulating age-related changes in neurotransmitter gene expression, with patterns that differ between neurotransmitter systems. GABAergic genes show consistent promoter hypermethylation across studies, which has been inversely associated with gene expression. In contrast, evidence of age-related methylation changes in dopaminergic and serotonergic genes during normative aging remains limited. This gap may reflect methodological challenges, including selective sampling of brain regions, cellular heterogeneity, and the limited use of cell type-specific approaches.

Histone modifications also appear to change with age, with shifts in activating and repressive marks reported at neurotransmitter-related loci. Gene ontology analyses have frequently identified enrichment of pathways related to neurotransmission. Advances in methodology, including single-nucleus multi-omics, targeted profiling of subcortical regions, and longitudinal study designs that combine peripheral biomarkers with neuroimaging, will be important for further progress in this field.

Clarifying whether epigenetic alterations contribute directly to age-related declines in neurotransmitter function and whether these mechanisms can be targeted therapeutically will require the integration of human observational studies with mechanistic work in model systems and well-designed intervention trials. Additionally, sex-related differences in epigenetic aging rates and neurotransmitter system trajectories remain incompletely characterized in the human brain and represent an important source of biological variation that warrants explicit consideration in future studies. As epigenetic regulation remains dynamic throughout the lifespan, it may offer opportunities for interventions aimed at preserving neurotransmitter function and cognitive health during aging.

## Figures and Tables

**Table 1:** Search Strategy.

Database	Search String
PubMed	(“aging”[MeSH Terms] OR “aging”[Title/Abstract] OR “ageing”[Title/Abstract] OR “senescence”[MeSH Terms] OR “senescence”[Title/Abstract] OR “biological aging”[Title/Abstract] OR “biological ageing”[Title/Abstract] OR “age-related”[Title/Abstract] OR “age related”[Title/Abstract] OR “lifespan”[MeSH Terms] OR “lifespan”[Title/Abstract] OR “life span”[Title/Abstract] OR “longevity”[MeSH Terms] OR “longevity”[Title/Abstract] OR “hallmarks of aging”[Title/Abstract] OR “hallmarks of ageing”[Title/Abstract]) AND (“epigenesis, genetic”[MeSH Terms] OR “epigenetic*”[Title/Abstract] OR “DNA methylation”[MeSH Terms] OR “DNA methylation”[Title/Abstract] OR “histone*”[Title/Abstract] OR “histone code”[MeSH Terms] OR “chromatin”[MeSH Terms] OR “chromatin remodeling”[Title/Abstract] OR “chromatin modification*”[Title/Abstract] OR “CpG islands”[MeSH Terms] OR “CpG”[Title/Abstract] OR “acetylation”[Title/Abstract] OR “deacetylation”[Title/Abstract] OR “methylation”[Title/Abstract] OR “demethylation”[Title/Abstract] OR “histone methylation”[Title/Abstract] OR “histone acetylation”[Title/Abstract] OR “H3K4me3”[Title/Abstract] OR “H3K27me3”[Title/Abstract] OR “H3K9me3”[Title/Abstract] OR “H4K20me3”[Title/Abstract] OR “microRNA*”[MeSH Terms] OR “miRNA*”[Title/Abstract] OR “miR-“[Title/Abstract] OR “non-coding RNA”[Title/Abstract] OR “ncRNA”[Title/Abstract] OR “long noncoding RNA”[MeSH Terms] OR “lncRNA”[Title/Abstract] OR “epigenetic clock*”[Title/Abstract] OR “epigenetic age”[Title/Abstract] OR “DNA methylation age”[Title/Abstract] OR “DNAm age”[Title/Abstract] OR “Horvath clock”[Title/Abstract] OR “Hannum clock”[Title/Abstract] OR “DNMT*”[Title/Abstract] OR “DNA methyltransferase*”[Title/Abstract] OR “TET enzyme*”[Title/Abstract] OR “histone deacetylase*”[Title/Abstract] OR “HDAC*”[Title/Abstract] OR “histone acetyltransferase*”[Title/Abstract] OR “HAT”[Title/Abstract]) AND (“neurotransmitter agents”[MeSH Terms] OR “neurotransmitter*”[Title/Abstract] OR “dopamine”[MeSH Terms] OR “dopamine”[Title/Abstract] OR “dopaminergic”[Title/Abstract] OR “serotonin”[MeSH Terms] OR “serotonin”[Title/Abstract] OR “5-HT”[Title/Abstract] OR “5-hydroxytryptamine”[Title/Abstract] OR “serotonergic”[Title/Abstract] OR “norepinephrine”[MeSH Terms] OR “norepinephrine”[Title/Abstract] OR “noradrenaline”[Title/Abstract] OR “noradrenergic”[Title/Abstract] OR “acetylcholine”[MeSH Terms] OR “acetylcholine”[Title/Abstract] OR “cholinergic”[Title/Abstract] OR “ACh”[Title/Abstract] OR “GABA”[Title/Abstract] OR “gamma-aminobutyric acid”[Title/Abstract] OR “GABAergic”[Title/Abstract] OR “gamma aminobutyric acid”[MeSH Terms] OR “glutamate”[Title/Abstract] OR “glutamic acid”[MeSH Terms] OR “glutamatergic”[Title/Abstract] OR “monoamine*”[Title/Abstract] OR “catecholamine*”[MeSH Terms] OR “catecholamine*”[Title/Abstract] OR “neurotransmission”[Title/Abstract] OR “synaptic transmission”[MeSH Terms] OR “neurotransmitter synthesis”[Title/Abstract] OR “neurotransmitter metabolism”[Title/Abstract] OR “neurotransmitter receptor*”[Title/Abstract] OR “receptor*”[Title/Abstract] OR “neurotransmitter transporter*”[Title/Abstract] OR “transporter*”[Title/Abstract] OR “excitatory”[Title/Abstract] OR “inhibitory”[Title/Abstract] OR “excitation inhibition balance”[Title/Abstract] OR “E/I balance”[Title/Abstract]) AND (brain OR cortex OR cortical OR CNS OR “central nervous system” OR neural OR neuron* OR “nervous system”) AND (“Caenorhabditis elegans”[MeSH Terms] OR “C. elegans”[Title/Abstract] OR “C elegans”[Title/Abstract] OR “nematode*”[Title/Abstract] OR “mice”[MeSH Terms] OR “mouse”[Title/Abstract] OR “mice”[Title/Abstract] OR “murine”[Title/Abstract] OR “Mus musculus”[Title/Abstract] OR “rats”[MeSH Terms] OR “rat”[Title/Abstract] OR “rats”[Title/Abstract] OR “Rattus”[Title/Abstract] OR “rodent*”[Title/Abstract] OR “Drosophila”[MeSH Terms] OR “Drosophila”[Title/Abstract] OR “fruit fly”[Title/Abstract] OR “fruit flies”[Title/Abstract] OR “zebrafish”[MeSH Terms] OR “zebrafish”[Title/Abstract] OR “Danio rerio”[Title/Abstract] OR “animal model*”[Title/Abstract] OR “model organism*”[Title/Abstract] OR “translational”[Title/Abstract] OR “cross-species”[Title/Abstract] OR “comparative”[Title/Abstract] OR “humans”[MeSH Terms]) AND (“cognition”[MeSH Terms] OR “cognitive decline”[Title/Abstract] OR “cognitive function”[Title/Abstract] OR “cognitive aging”[Title/Abstract] OR “neurodegenerative diseases”[MeSH Terms] OR “neurodegeneration”[Title/Abstract] OR “Alzheimer*”[Title/Abstract] OR “Parkinson*”[Title/Abstract] OR “neuropsychiatric”[Title/Abstract] OR “mood disorder*”[Title/Abstract] OR “depression”[MeSH Terms] OR “anxiety”[MeSH Terms] OR “brain aging”[Title/Abstract] OR “brain age”[Title/Abstract] OR “neural”[Title/Abstract] OR “neuronal”[Title/Abstract])
Embase	(‘aging’/exp OR ‘senescence’/exp OR ‘aging’:ti,ab OR ‘ageing’:ti,ab OR ‘age-related’:ti,ab OR ‘age related’:ti,ab) AND (‘epigenetics’/exp OR ‘DNA methylation’/exp OR ‘histone modification’/exp OR ‘chromatin’/exp OR ‘microRNA’/exp OR ‘epigenetic*’:ti,ab OR ‘DNA methylation’:ti,ab OR ‘histone*’:ti,ab OR ‘chromatin remodeling’:ti,ab OR ‘histone modification*’:ti,ab OR ‘epigenetic clock*’:ti,ab OR ‘DNAm age’:ti,ab OR ‘miRNA’:ti,ab OR ‘histone acetylation’:ti,ab OR ‘histone methylation’:ti,ab) AND (‘neurotransmitter’/exp OR ‘dopamine’/exp OR ‘serotonin’/exp OR ‘noradrenaline’/exp OR ‘acetylcholine’/exp OR ‘gamma aminobutyric acid’/exp OR ‘glutamic acid’/exp OR ‘neurotransmitter*’:ti,ab OR ‘dopamine’:ti,ab OR ‘dopaminergic’:ti,ab OR ‘serotonin’:ti,ab OR ‘serotonergic’:ti,ab OR ‘5-HT’:ti,ab OR ‘norepinephrine’:ti,ab OR noradrenaline’:ti,ab OR ‘noradrenergic’:ti,ab OR ‘acetylcholine’:ti,ab OR ‘cholinergic’:ti,ab OR ‘GABA’:ti,ab OR ‘GABAergic’:ti,ab OR ‘gamma aminobutyric’:ti,ab OR ‘glutamate’:ti,ab OR ‘glutamatergic’:ti,ab OR ‘neurotransmission’:ti,ab OR ‘synaptic transmission’:ti,ab)
PsycINFO	exp Aging/OR exp Age Differences/OR exp Senescence/ AND (DNA Methylation/OR Epigenetics/OR Histones/OR Chromatin/) AND (exp Neurotransmitters/OR exp Dopamine/OR exp Serotonin/OR exp Norepinephrine/OR exp Acetylcholine/OR exp GABA/OR exp Glutamic Acid/OR exp Neurotransmission/)

**Table 2:** Inclusion and Exclusion Criteria.

Category	Inclusion Criteria	Exclusion Criteria
Study focus	Studies examining epigenetic regulation in relation to neurotransmitter systems	Studies on epigenetics or aging without neurotransmitter relevance
Epigenetic scope	DNA methylation, histone modifications, chromatin, epigenetic clocks	Studies without epigenetic mechanisms
Neurobiological scope	Neurotransmitter systems	Non-neurotransmitter molecular pathways only
Population	Human studies, comparative studies including humans	Animal model-only studies, in vitro-only studies, case reports
Outcomes	Neurobiological, cognitive, or neuropsychiatric relevance	No functional, clinical, or neural relevance
Tissue context	Brain, CNS, or neural systems	Peripheral-only studies without clear neural relevance
Study type	Original research, reviews	Editorials, conference abstracts

**Table 3:** Major DNA Methylation Clocks Relevant to Brain Aging.

Clock	Year	CpG Sites	Training Tissue(s)	Primary Outcome	Key Features	Reference
Horvath	2013	353	Pan-tissue (51 types)	Chronological age	High pan-tissue accuracy (r = 0.96); enriched at developmental genes	[10]
Hannum	2013	71	Whole blood	Chronological age	Reveals genetic/sex effects on aging rate	[9]
DNAmClockCortical	2020	347	Brain cortex	Chronological age	Improved accuracy in aged brain; region-specific	[27]
DNAm PhenoAge	2018	513	Whole blood	Phenotypic age, mortality	Predicts mortality, cancer, physical function, and AD.	[11]
GrimAge	2019	1030	Whole blood	Lifespan, healthspan	Strongest mortality predictor; includes smoking, plasma proteins	[25]
DunedinPACE	2020	173	Whole blood	Pace of aging	Measures rate of aging from longitudinal data	[13]

**Table 4:** Histone Modifications at Neurotransmitter-Related Loci in Aging Brain.

Histone Mark	Modification Type	Age/Disease-Related Change	Genomic Context	Associated Pathways	Ref.
H4K16ac	Acetylation (activating)	↑ in normal aging; ↓↓ in AD	Promoters, enhancers	Aging genes, AD risk loci	[31]
H3K27ac	Acetylation (activating)	↑ at chromatin regulators in AD	Promoters	Transcriptional regulation	[32]
H3K9ac	Acetylation (activating)	Variable changes	Gene bodies	Chromatin remodeling	[32]
H3K4me3	Methylation (activating)	↓ at synaptic/NT genes; ↑ at transcriptional regulators	Promoters	Neurotransmission, synaptic plasticity	[33,34]
H3K27me3	Methylation (repressive)	Altered at developmental genes	Promoters, intergenic	Polycomb targets	[33]
H4K16ac	Acetylation (activating)	↑ in normal aging; ↓↓ in AD	Promoters, enhancers	Aging genes, AD risk loci	[31]

Arrows highlight the directionality of the changes mentioned where ↑ highlights an increase in the related change and ↓ denotes a decrease in the related change.

**Table 5:** Age-Related DNA Methylation Changes at Neurotransmitter Genes in Human Brain.

Gene	Protein/Function	Brain Region(s)	Age-Related Change	Expression Correlation	Study Population	Ref.
*GAD1*	GAD67 (GABA synthesis)	Temporal cortex, PFC	Progressive ↑ methylation	Inverse (R^2^ = 0.29–0.42)	*n* = 125, fetal-87 years	[38,39]
*GABRA2*	GABA-A receptor α2	PFC	↑ methylation	Inverse correlation	*n* = 108, fetal-90 years	[38]
*DRD2*	Dopamine D2 receptor	PFC	Modest ↑ methylation	Not extensively characterized	*n* = 108, fetal-90 years	[38]
*SLC6A4*	Serotonin transporter	Blood, PFC (limited)	Variable; genotype-dependent	Conflicting peripheral-brain associations	Multiple cohorts	[45,47]
*TPH2*	Tryptophan hydroxylase 2	Blood (brain data sparse)	No clear association with brain 5-HT in PET studies	No association	*n* = 344	[44]
*GRIN2B*	NMDA receptor NR2B	Multiple brain regions	H3K4me3 changes (histone, not DNAm)	Region-specific patterns	Multiple	[34]

Arrows highlight the directionality of the changes mentioned where ↑ highlights an increase in the related change.

## Data Availability

The search strategies used in this review are presented in Table 1 of the manuscript. No additional datasets were generated or analyzed beyond those reported in the included studies.
